# Ethnoecology of the interchange of wild and weedy plants and mushrooms in Phurépecha markets of Mexico: economic motives of biotic resources management

**DOI:** 10.1186/s13002-018-0205-z

**Published:** 2018-01-15

**Authors:** Berenice Farfán-Heredia, Alejandro Casas, Ana I. Moreno-Calles, Eduardo García-Frapolli, Aída Castilleja

**Affiliations:** 1 Universidad Intercultural Indígena de Michoacán, Finca “La Tsípecua” kilómetro 3 carretera, Pátzcuaro-Huecorio, Michoacán C.P. 61614 México; 2 Instituto de Investigaciones en Ecosistemas y Sustentabilidad, UNAM, Antigua Carretera a Pátzcuaro 8711, Morelia, Michoacán 58190 México; 3 Escuela Nacional de Estudios Superiores, Unidad Morelia, Universidad Nacional Autónoma de México (UNAM), Morelia, Michoacán 58190 México; 4 Instituto Nacional de Antropología e Historia, Insurgentes Sur No. 421, Colonia Hipódromo, México D.F, CP 06100 México

**Keywords:** Ethnoecology, Traditional markets, Non-crop resources, Interchange, Barter, Phurépecha culture, Plant management, Non-timber forest resources management

## Abstract

**Background:**

Interactions between societies and nature are regulated by complex systems of beliefs, symbolism, customs, and worldviews (*kosmos)*, ecological knowledge (*corpus*), and management strategies and practices (*praxis*), which are constructed as product of experiences and communication of people throughout time. These aspects influence social relations, life strategies, and cultural identity, and all of them in turn influence and are influenced by local and regional patterns of interchange. In this study, we analyze the interchange of wild and weedy plants and mushrooms in traditional markets of the Phurépecha region of Mexico. Particularly, the social relations constructed around the interchange of these products; how knowledge, cultural values, and ecological factors influence and are influenced by interchange; and how all these factors influence the type and intensity of biotic resources management.

**Methods:**

We studied three main traditional markets of the Phurépecha region of Michoacán, Mexico, through 140 visits to markets and 60 semi-structured interviews to sellers of wild and weedy plants and mushrooms. In nearly 2 years, we carried out 80 visits and 30 interviews in the “Barter Market”, 20 visits and 15 interviews in the “Phurépecha Tiánguis”, and 40 visits and 15 interviews to the “Municipal Market”. We documented information about the spaces of interchange that form the markets, the types of interchange occurring there, the cultural and economic values of the resources studied, the environmental units that are sources of such resources, the activities associated to resources harvesting and, particularly, the management techniques practiced to ensure or increase their availability. We analyzed the relations between the amounts of products interchanged, considered as pressures on the resources; the perception of their abundance or scarcity, considered as the magnitude of risk in relation to the pressures referred to; and the management types as response to pressures and risk.

**Results:**

We recorded 38 species of wild and weedy plants and 15 mushroom species interchanged in the markets. We characterized the spaces of interchange, the interchange types, and social relations among numerous Phurépecha communities which maintain the main features of pre-Columbian markets. The products analyzed are differentially valued according to their role in people’s life, particularly food, medicine, rituals, and ornamental purposes. The highest cultural values were identified in multi-purpose plant and mushroom resources and, outstandingly, in ornamental and ritual plants. In markets, women are the main actors and connectors of the regional households’ activities of use and management of local resources and ecosystems. The interrelationships between worldviews, knowledge, and practices are visible through the interchange of the products analyzed, including the types of environments comprised in communitarian territories, agricultural calendars, and feasts. Those plants and mushrooms are highly valued but relatively scarce according to the demand on them receiving special attention and management practices directed to ensure or increase their availability. With the exception of most mushrooms and ornamental and ritual plants, which have high economic and cultural values, there are those that are relatively scarce and under high risk, but are obtained through simple gathering from the wild.

**Conclusions:**

Traditional markets are crucial part of the subsistence strategy of Phurépecha people based on the multiple use of resources and ecosystems at the local and regional levels. The markets influence social relations, cultural identity, and preservation of traditional knowledge and biodiversity. In general, the demand of products in markets enhances innovation and practices for ensuring or increasing their availability, particularly those that are naturally scarce. However, it was notorious that, althoug mushrooms and ritual plants have high demand and value in markets, most of them are obtained by simple gathering.

## Background

The form in which human societies interact with nature is influenced by complex systems of symbolic elements, customs, beliefs, and worldviews (the *kosmos*), traditional ecological knowledge (TEK, or *corpus*), and management practices and strategies (the *praxis*) [1–3]. The worldview systems are constructed based on the form of how people perceive, interpret, and explain their surrounding social and natural contexts, which influence their social life and relation with their territories. These systems reflect the conceptions and representations of the space, time, the notion of belonging to a cultural group, strategies of social organization, rituals, forms of transmission of knowledge, and practices of production [4–9]. TEK is represented by the body of experiences and conceptualizations resulting from the coexistence of a cultural group with nature; it conforms to the traditional cognitive system for recognizing, systematizing, classifying, and relating the elements of nature by that group [1, 5]. The management techniques conforming to the *praxis* include the diversity of strategies, planning, practices or forms of interaction for transforming, conserving, recovering, or adapting ecosystems to human views, needs, and purposes. Such practices are carried out with different levels of intensity according to the role both ecosystems and resources play in peoples’ life [10–13].

TEK and management strategies and practices are intimately connected with the system of worldview [1, 5, 13]. The interpretations, representations, and forms of appropriation of nature at different spatial and temporal scales are therefore included in what is called by several authors the *kosmos-corpus-praxis* complex [3, 5, 14], in which interaction to local ecosystems and resources, determining particular life strategies and cultural identity [5, 9, 13, 15].

As part of life strategies, the traditional management strategies are based on the diversification of production by households, as well as on the interchange of products, all of which favors the diversified access to useful goods for complementing subsistence [16–18]. The interchange can therefore be considered the node of a net of management actions, which may influence the type and intensity of management of natural resources and ecosystems according to values, cultural significance, and demand and scarcity of products in the interchange contexts [11, 12, 19, 20]. Through processes of interchange, products derived from management strategies by some people are available to other people, a feature characteristic of numerous cultural groups in Mesoamerica. Through interchange, social relations are constructed, and the products’ values result from their meaning, social function and importance in people’s life [12, 20, 21].

The traditional Mesoamerican markets still exist in some regions and form important part of the regional cultures and social relations of peoples of the area. Among others, these markets have the following features: (1) have pre-Columbian origin and maintain aspects of their physiognomy, (2) involve different forms of interchange, including barter, (3) are temporary, commonly weekly, (4) people of different ethnic groups have a setting of cultural interaction, (5) predominate the active role of women, and (6) coexist and interact with conventional modern markets [20, 22]. In traditional markets, it is common to find wild plants, animals, and mushrooms, as well as weedy plants products, which are obtained in homegardens, hunted or gathered in forests, and collected in ruderal and agricultural areas. These products still have high economic and cultural importance for subsistence of the rural households [18, 21, 22].

Our study analyses the processes of interchange of wild and weedy plants and mushrooms in the main traditional Phurépecha markets, in Michoacán, central Mexico. We particularly investigated the social, cultural, and economic relations associated with the interchanged products referred to, their relation to the traditional knowledge, their cultural values, and the social organization to obtain and interchange them. We particularly emphasized the relation of such context with practices of traditional management to ensure their availability to satisfy the needs of interchange. We previously have explored how management practices constitute responses to the need of ensuring availability of resources [11, 13, 20]. Such needs of availability may be influenced by their distribution and abundance and the magnitude of their importance in people’s life. These aspects determine balances between what is available and what is needed by a human group, involving ecological, cultural, and economic aspects. Interchange is an old strategy practiced by humans to attend the challenge of ensuring availability of some resources, but at the same time it establishes the need of making available for other people what some peoples have. And such situation may determine increase, in some contexts, and/or decrease, in others, of pressure on important resources [11, 13, 20]. We have hypothesized that those situations that increase pressure on valuable resources commonly influence management decisions, which may be more intense according to the magnitude of pressure and the risk associated to low availability of products and their high demand. Therefore, the interchange may be crucial for understanding the motivations of management of biotic resources. Our main premise of the current study is that markets are areas where the relations of the complex *kosmos-corpus-praxis* are expressed as part of the life strategies and the cultural identity of human groups. Therefore, we look into analyzing how interchange influences the need of management resources and ecosystems. We particularly explored the hypothesis that those resources with higher demand and interchange value in markets, but scarce in forests or other sources of resources provenance, enhance management practices. We conducted this study mainly from qualitative perspectives among the Phurépecha people of Michoacán, analyzing how such interchange relations in barter and trade in the main markets of the Phurépecha and how the intensity of these forms of interchange, in terms of supply and demand, influence management intensity.

## Methods

### Study area

**Fig. 1:**
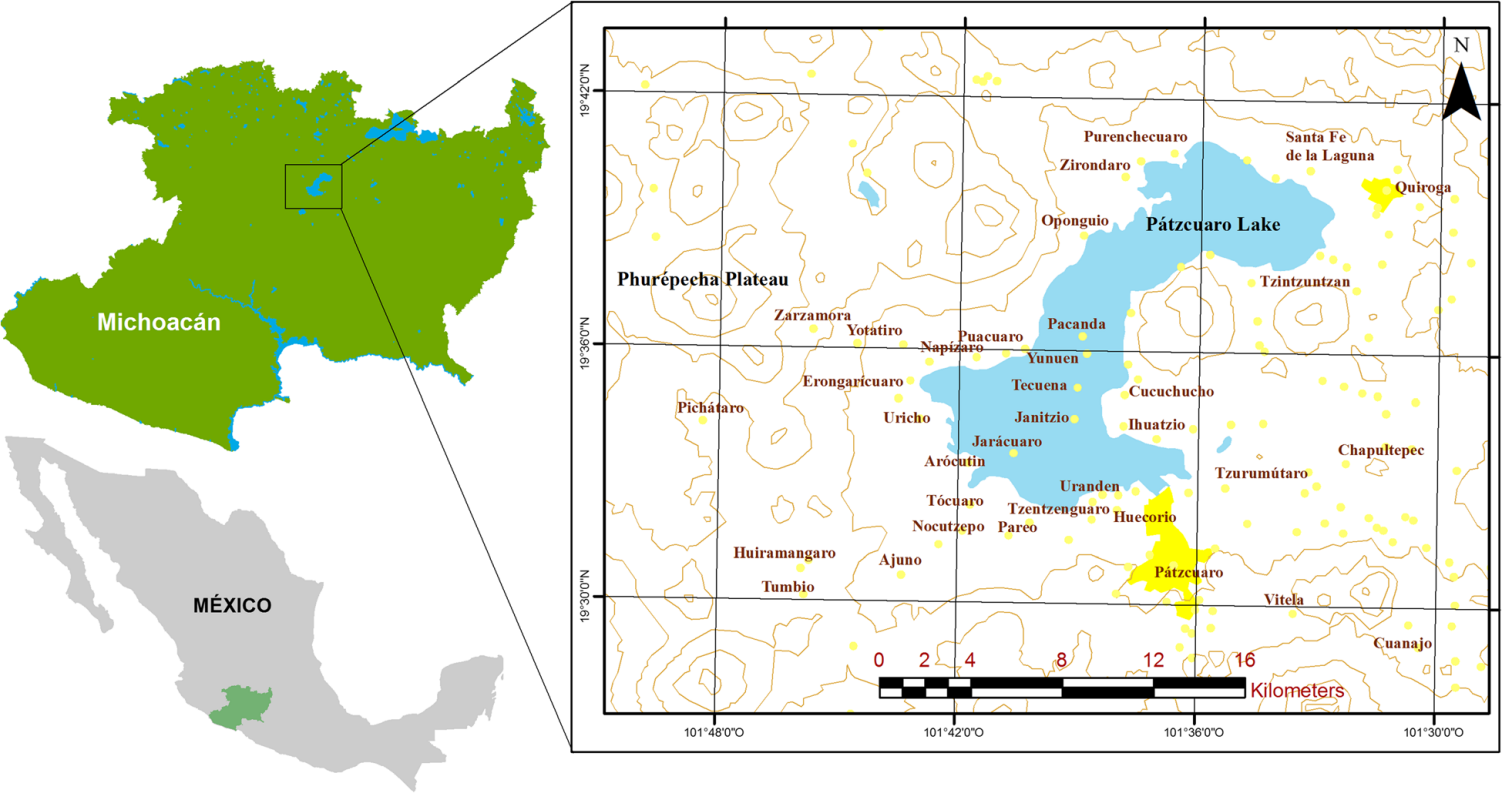
Study area. Location of the regions of the Pátzcuaro Lake and the Phurépecha Plateau, in the state of Michoacán, México. Cities and communities referred to in the main text

The regions of the Pátzcuaro Lake and the Phurépecha Plateau in the state of Michoacán, central Mexico, are the main territory of the Phurépecha people. This region is located in the Neovolcanic transversal belt crossing central Mexico, with mountains, plains, rolling hills, and valleys in elevations ranging from 2100 to 3280 m (Fig. 1). Climate is temperate sub-humid with summer rains. Vegetation is predominantly oak and pine forests, with patches of subtropical scrub and aquatic vegetation in lakes [23].

The economy of the local people is based on irrigated and seasonal agriculture, fruit perennial crops, extensive livestock, silvicultural practices, fishing, and elaboration of handicrafts. Because of its heterogeneous ecosystems and biocultural diversity, the region offers a high variety of utilitarian products influencing strategies of cultural diversification or multiple use of the territories of the communities [21, 24].

### Markets studied

**Fig. 2:**
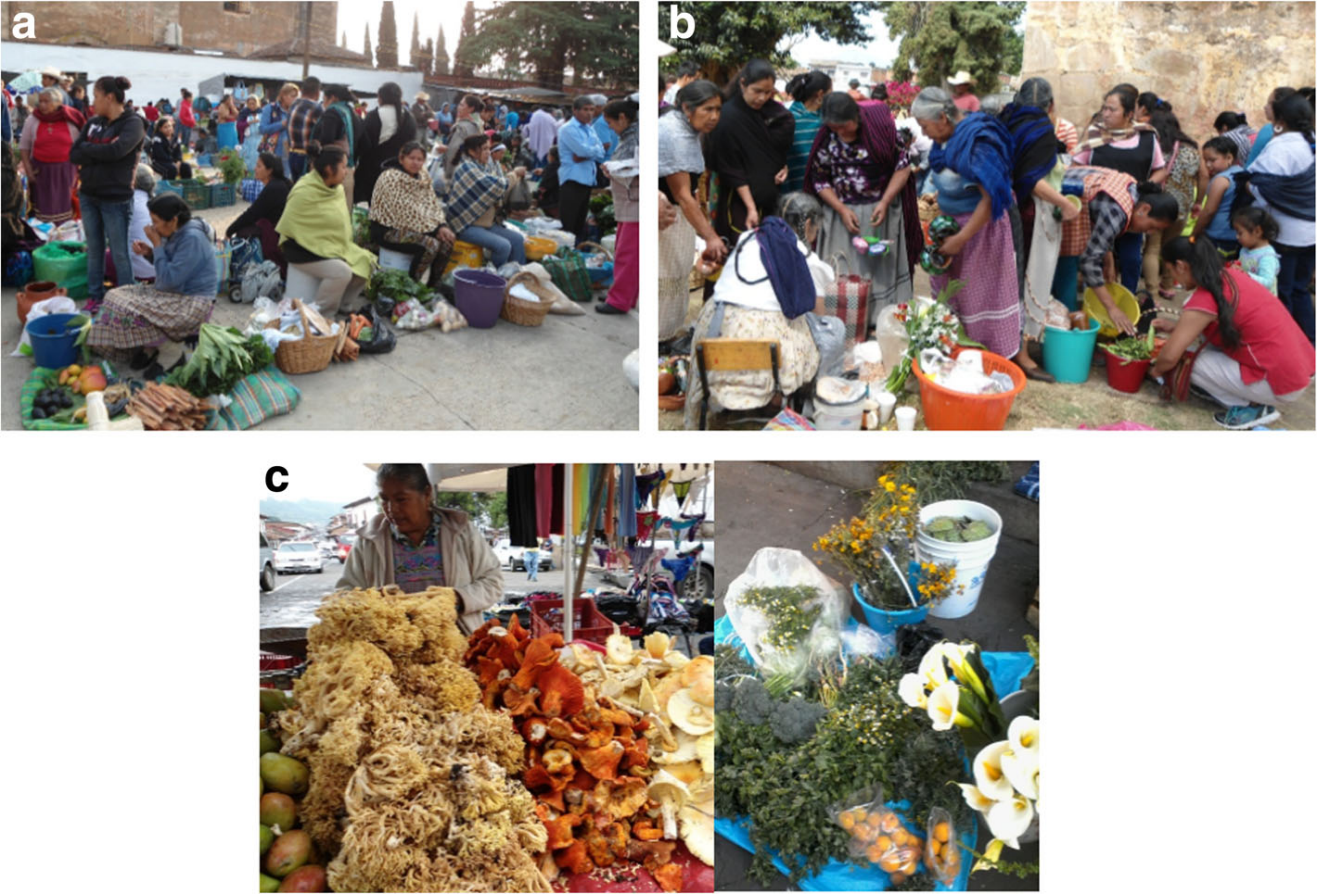
Aspects of the markets studied **a** Barter Market in the city of Pátzcuaro, **b** Phurépecha Tiánguis, the regional *Mojtakuntani* itinerant in several communities of the Pátzcuaro Lake shoreline, and **c** Municipal Market of the city of Pátzcuaro

We studied the “Municipal Market,” the main established market of the city of Pátzcuaro. There, some permanent stands are part of the infrastructure, but temporary informal sellers arrive every day, occupying the surrounding streets of the market, thus complementing the configuration of the market. Monetary interchange is predominant in this market, but barter transactions are common among people established in the surrounding areas. We in addition studied the “Mercado de Cambio,” also called the barter market (ahead called the “Barter Market”), which is also located in the city of Pátzcuaro, where interchangers from 42 communities of the Pátzcuaro Lake Region participate, and it is carried out twice per week (Tuesday and Friday). There, people interchange products processed at home, utilitarian and handicraft objects, and products derived from agriculture, extracted from forests, gathered, and fished [25]. Participants are Phurépecha and Mestizo people from rural communities of the region (Fig. 2).

Another market studied was the regional “Phurépecha Tiánguis” founded in 1994, also called *mojtakuntani*, which means “interchanging like brothers” in Phurépecha, which promotes actions directed to enhance reciprocity, traditional production, and barter [26]. It is carried out twice per week, every Sunday, rotating from place to place among the 15 communities participating in it. In this market, products of agriculture and forests, fishing, tools, and handicraft are mainly supplied (Fig. 2). Participants are predominantly Phurépecha and Mestizo peoples [26, 27].

We conducted interviews to producers, gatherers, and sellers in the market who obtain wild and weedy plant resources for interchange in different seasons. We finally included sellers in the market who buy their products from producers and gatherers (Fig. 2).

### Data collection

Through participant observations and semi-structured interviews in the three markets studied, we recorded main aspects of interchange. Particularly, we documented information about the characteristics of the spaces of interchange, the provenance and ethnicity of people participating in the markets, and the types of interchange. We centered our attention in documenting ecological, economic, and management practices associated to wild and weedy plant resources, as well as the mushrooms interchanged in these markets. We emphasized obtaining information on the forms of use of these resources, their spatial (types of environmental units where they are harvested) and seasonal availability, the management practices and strategies that people put in practice in order to ensure or increase their availability, and the details about the type of interchange these species involve. We carried out 80 visits and 30 semi-structured interviews to sellers of wild and weedy plants and mushrooms in the Barter Market, 20 visits and 15 interviews to sellers of wild and weedy plants and mushrooms in the Phurépecha Tiánguis, and 40 visits and 15 interviews in the Municipal Market. All these activities were conducted from February 2015 to November 2016.

Botanical samples of plants and fungi were collected, and photographic records were made of fruits, fungi, cladodes, orchids, and products that did not comply with the conventional characteristics of proper botanical specimen. Nomenclature and classification of plant species reported followed the APG III classification system reviewed in the site www.theplantlist.org. Scientific names of mushrooms were consulted in the Index fungorum: http://www.indexfungorum.org.

## Results

### Spaces of interchange

Because the Barter Market is derived from the pre-Columbian Phurépecha markets from the cities of Tzintzuntzan and Pareo [17, 28], this market has been practiced in several communities throughout time. At present, it is settled in the City of Pátzcuaro, but it is not a site with permanent infrastructure. Rather, it is a space of interchange constructed through social relations of people participating in it, the type of products offered, and the interest of maintaining the custom of offering and obtaining something.

In the Phurépecha Tiánguis, the space of interchange is constructed based on the social relations among the communities participating, in order to make possible a space of cohabitation and communal living, for offering and interchanging products, and maintaining the custom of bartering. Interchange is carried out in public spaces of communities that participate. The particularity of this market is that the community that becomes host every week offers merchants from other communities a welcome midday meal with music and chants in Phurépecha.

The Municipal Market has a permanent infrastructure; however, numerous informal stands are established in the streets surrounding the market. The producers, gatherers, and sellers lack a fixed place, thus they offer their products in spaces available in the streets or inside the main market.

### Form of exhibiting the interchange products and people participating in markets

In the three markets studied, the producers, gatherers, and sellers put their wild and weedy products on clothes or plastic pieces placed on the floor, in baskets, buckets, or woody boxes, whereas people that have formal places in markets exhibit wild and weedy products in stacks placed apart from the cultivated products (Fig. 2).

In the three markets analyzed, people offering wild and weedy products are mainly local peasants, gatherers, artisans, and fishermen. In the Barter Market, people participate from 29 rural communities of the region, while in the Municipal Market, people participate from 17 communities, and in the Phurépecha Tiánguis, from 5 communities. The Barter Market, as well as in the Municipal Market, may additionally have participation of sellers from urban or other rural areas of the region, who re-sell wild and weedy plant resources in other regions of the state of Michoacán and Mexico City.

The number of people participating every day in the Barter Market averaged 174 ± 34, and nearly 4.8% of them offer wild and weedy plants and mushrooms. In the Phurépecha Tiánguis, an average 28 ± 6 of people participate every day, 5.5% of them selling wild and weedy plants and mushrooms, whereas in the Municipal Market, an average of 50 ± 27 people participate per day, 5% of them selling wild and weedy plants and mushrooms.

The participation of plants and mushrooms sellers is variable throughout the year. Some of them interchange products specific for the particular seasons and communities. For instance, the capulines (*Prunus serotina*) from the community of San Juan Tumbio and San Francisco Pichátaro are available from April to June. Sellers of mushrooms from Cuanajo, San Francisco Pichátaro, Ajuno, Zirahuén, Santa Ana, Pátzcuaro, and Huecorio can be found from June to August. On the other hand, the ornamental and ceremonial orchids (*Laelia speciosa* and *L. autumnalis*) and other flowers with similar purposes are sold at most 1 month throughout different seasons of the year.

Other sellers go to the markets the whole year offering several types of products. For instance, women from Cuanajo offer medicinal plants like marrubio (*Marrubium vulgare*), toronjil (*Agastache mexicana*), té nurite (*Clinopodium macrostemum*), árnica (*Heterotheca inuloides*), gordolobo (*Gnaphalium* sp.), and hierba del cáncer (*Acalypha phleoides*), throughout the year. These plants are collected in forests, ruderal areas, agricultural fields, and homegardens and are sold in markets together with seasonal products. The number of sellers in markets is extraordinarily variable throughout the year, according to the seasonality of wild products, agricultural harvesting times, and religious ceremonies.

### Interchange types

In the Barter Market, wild and weedy plant resources are interchanged through barter and monetary interchange. Their value is established according to cultural, economic, and utilitarian considerations. For instance, bunches of flowers from cultivated, wild, or weedy plants are exclusively destined to monetary interchange, because of their high value for ceremonial and ornamental purposes among the Phurépecha people. Some weedy plants like the traditional greens generically called “quelites” in Náhuatl or “*xakua*” in Phurépecha, among them the species called Juan Primero (*Rumex obtusifolius*) and the “quelite de trigo” (*Amaranthus hybridus*), are interchanged through barter or in some cases by trade at low price, because of their slightly bitter flavor. Wild plants with medicinal use like the “hierba del cáncer” (*Acalypha phleoides)* and the “istafiate” (*Artemisia ludoviciana*) are interchanged through barter or by selling at low prices because their use is uncommon. The type of interchange of edible mushrooms depend on their quality; entire mushrooms are destined to monetary interchange, while broken mushrooms are bartered (Tables 1, 2, and 3). Considering the number of people participating in the Barter Market, the interchange intensity through barter is higher than that in the other two markets, and although this market occurs 2 days per week, the variety and amounts of wild and weedy plants and mushrooms interchanged is high. This market contributes significantly to the provision of resources of a high number of households of the region.

**Table 1:** Wild and weedy plants interchanged in the traditional Phurépecha markets studied

Scientific name	Common name	Use form	Distribution area	Management form	Market*	Interchange type**	Offering communities	Months of the year offered	Voucher number
*Agave inaequidens* Koch	Jiote	Edible	Pine-oak and oak forests	Gathering	1, 3	Barter (trade)	3	7	PhR
*Amaranthus hybridus* L.	Quelite de trigo, quintonil	Edible	Agricultural areas	Gathering, tolerance	1, 3	Barter, trade	4	6	BFH-362
*Brassica rapa* L.	Mostaza (vaina y quelite)	Edible	Agricultural areas	Gathering, tolerance	1, 2, 3	Barter, trade	5	7	BFH-360
*Chenopodium berlandieri* Moq.	Quelite cenizo	Edible	Lake shoreline, agricultural areas	Gathering, enhancing	1, 2, 3	Barter, trade	10	11	BFH-351
*Crataegus mexicana* Moc. & Sessé ex DC	Tejocote	Edible	Pine-oak and oak forests, agricultural areas	Gathering	1, 2	Barter	1	3	PhR
*Opuntia atropes* Rose	Nopales	Edible	Subtropical scrub, homegardens	Gathering, enhancing	1, 2, 3	Barter, trade	15	10	PhR
*Opuntia* sp.	Xoconostle	Edible	Subtropical scrub	Gathering	1	Barter (trade)	1	3	PhR
*Portulaca oleracea* L.	Verdolaga	Edible	Agricultural areas	Gathering, tolerance, enhancing	1, 3	Barter, trade	10	6	BFH-365
*Prunus serotina* subsp. *capuli* (Cav. ex Spreng.) McVaugh	Capulines (fruits and flowers)	Edible	Pine-oak and oak forests, agricultural areas, homegardens	Selective gathering, selective tolerance, selective enhancing	1, 2, 3	Barter, trade	6	4	PhR
*Rorippa nasturtium-aquaticum* (L.) Hayek	Berro	Edible	Lake shoreline	Gathering	1, 3	Barter, trade	4	8	BFH-355
*Rubus Liebmannii* Focke	Zarzamora	Edible	Pine-oak and oak forests	Gathering	1, 2, 3	Trade	7	8	PhR
*Rumex obtusifolius* L.	Juan Primero	Edible	Agricultural areas, ruderal areas, pine-oak and oak forests	Gathering	1, 2	Barter	7	8	BFH-359
*Solanum lycopersicum* L.	Jitomate silvestre	Edible	Agricultural areas	Gathering, enhancing	2	Barter	1	1	PhR
*Tagetes micrantha* Cav.	Anís	Edible, condiment	Agricultural areas, ruderal, pine-oak and oak forests	Selective gathering	1, 3	Barter, trade	10	7	BFH-366
*Dysphania ambrosioides* (L.) Mosyakin & Clemants	Epazote	Edible as condiment	Homegardens	Gathering, enhancing	1, 2, 3	Trade, barter	4	5	BFH-361
*Laelia autumnalis* (Lex.) Lindl.	Flor de ánima o lirio	Ceremonial	Pine-oak and oak forests	Gathering	1, 3	Trade	2	1	PhR
Bryophyta sensu *lato*	Musgo	Ceremonial, ornamental	Pine-oak and oak forests	Gathering	3	Trade	–	2	PhR
*Calochortus purpureus* (Kunth) Baker	Flores moraditas	Ceremonial, ornamental	Pine-oak and oak forests	Gathering	1	Trade	2	1	BFH-371
*Castilleja scorzonerifolia* Kunth	Flor de terciopelo	Ceremonial, ornamental	Pine-oak and oak forests	Gathering	1	Trade	1	1	BFH-373
*Cosmos bipinnatus* Cav.	Mirasoles	Ceremonial, ornamental	Pine-oak and oak forests, agricultural areas, ruderal	Gathering	1, 3	Trade	1	1	BFH-372
*Laelia speciosa* (Kunth) Schltr.	Orquídea, flor de *corpus*	Ceremonial, ornamental	Pine-oak and oak forests	Gathering	1, 2, 3	Barter, trade	1	2	PhR
*Lupinus montanus* Kunth	Flor morada	Ceremonial, ornamental	Pine-oak and oak forests	Gathering	1	Trade	1	1	PhR
*Stevia monardifolia* Kunth	Servilletilla	Ceremonial, ornamental	Pine-oak and oak forests	Gathering	1	Trade	1	1	PhR
*Tillandsia* sp.	Heno	Ceremonial, ornamental	Pine-oak and oak forests	Gathering	3	Trade	–	2	PhR
*Milla biflora* Cav.	Estrellitas	Ceremonial, ornamental, medicinal, aromatic	Pine-oak and oak forests	Gathering	1, 3	Trade	4	2	BFH-370
*Tagetes lucida Cav.*	Santa María	Ceremonial, ornamental, medicinal, insecticide	Pine-oak and oak forests, ruderal, agricultural areas	Gathering	1	Trade	3	3	BFH-367
*Acalypha phleoides* Cav.	Hierba del cáncer	Medicinal	Homegardens	Enhancing	1	Barter, trade	1	1	BFH-363
*Agastache mexicana* (Kunth) Lint & Epling	Toronjil	Medicinal	Pine-oak and oak forests, homegardens	Gathering, enhancing	1, 2	Barter, trade	2	5	BFH-353
*Artemisia ludoviciana* Nutt.	Istafiate	Medicinal	Pine-oak and oak forests, ruderal	Gathering	1	Barter, trade	1	1	BFH-374
*Chenopodium graveolens* Lag & Rodr.	Epazote de perro	Medicinal	Homegardens	Gathering, enhancing	3	Trade	1	1	BFH-369
*Clinopodium macrostemum* (Moc. & Sessé ex Benth.) Kuntze	Nurite	Medicinal	Pine-oak and oak forests	Gathering, enhancing, transplanting, sowing	1, 2, 3	Barter, trade	2	5	BFH-354
*Equisetum* sp.	Cola de caballo	Medicinal	Riparian vegetation	Gathering	1, 3	Barter, trade	4	10	BFH-357
*Eryngium carlinae* F. Delaroche	Hierba del sapo	Medicinal	Pine-oak and oak forests, ruderal	Gathering	1, 3	Barter, trade	3	2	BFH-368
*Gnaphalium* spp.	Gordolobo	Medicinal	Pine-oak and oak forests, agricultural areas, ruderal	Gathering	1, 2, 3	Barter, trade	3	6	BFH-358
*Heterotheca inuloides* Cass.	Árnica	Medicinal	Pine-oak and oak forests, ruderal	Gathering	1, 3	Barter, trade	10	11	BFH-356
*Loeselia mexicana (*Lam.) Brand	Espinosilla	Medicinal	Pine-oak and oak forests	Gathering	1, 3	Trade	2	2	BFH-364
*Marrubium vulgare* L.	Marubio	Medicinal	Homegardens, ruderal	Gathering, enhancing	1, 2, 3	Barter, trade	3	11	BFH-352
*Ternstroemia lineata* DC.	Trompillo	Medicinal	Pine-oak and oak forests	Gathering	1, 2, 3	Trade	1	2	PhR

*Markets studied: *1* Barter Market, *2* Phurépecha Tiánguis, and *3* Municipal Market

**In parentheses the type of interchange less frequent

**Table 2:** Wild mushrooms interchanged in the Phurépecha traditional markets

Scientific name	Common name	Use form	Distribution area	Management form	Market type*	Interchange type	Offering communities	Offer per year	Voucher number
*Ramaria fenica* (P. Karst.) Ricken	Patitas de pájaro	Edible	Pine-oak and oak forests	Gathering	1, 3	Barter, trade	5	4	PhR
*Ramaria flavigelatinosa* Marr & D.E. Stuntz	Patitas de pájaro	Edible	Pine-oak and oak forests	Gathering	1, 2, 3	Barter, trade	5	4	BFH-H001
*Ramaria araiospora* Marr & D.E. Stuntz	Patitas de pájaro	Edible	Pine-oak and oak forests	Gathering	1, 3	Barter, trade	5	4	PhR
*Ramaria botrytis* (Pers.) Ricken	Patitas de pájaro	Edible	Pine-oak and oak forests	Gathering	1, 2, 3	Barter, trade	5	4	BFH-H002
*Ramaria flava* (Schaeff.) Quél.	Patitas de pájaro	Edible	Pine-oak and oak forests	Gathering	1, 2, 3	Barter, trade	5	4	BFH-H003
*Lyophyllum connatum* (Schumach.) Singer	Guachitas, pashacuas	Edible	Pine-oak and oak forests	Gathering	1, 3	Barter, trade	3	4	PhR
*Lyophyllum decastes* (Fr.) Singer	Guachitas, pashacuas	Edible	Pine-oak and oak forests	Gathering	1, 3	Barter, trade	2	4	PhR
*Agaricuas campestris* L.	Hongo llanero	Edible	Grasslands	Gathering	1, 3	Barter, trade	4	1	BFH-H004
*Amanita caesarea* (Scop.) Pers.	Hongo amarillo	Edible	Pine-oak and oak forests	Gathering	1, 3	Barter, trade	4	2	PhR
*Hypomyces lactifluorum* (Schwein.) Tul. & C. Tul.	Hongo trompa de puerco	Edible, condiment	Pine-oak and oak forests	Gathering	1, 3	Barter, trade	4	6	BFH-H005
*Calvatia cytahiformis* (Bosc) Morgan	Hongo globoso	Edible	Pine-oak and oak forests	Gathering	1	Barter, trade	1	1	PhR
*Helvella crispa* (Scop.) Fr.	Oreja de ratón blanca	Edible	Pine-oak and oak forests	Gathering	1	Barter, trade	1	1	BFH-H006
*Laccaria laccata* (Scop.) Cooke	Moradito	Edible	Pine-oak and oak forests	Gathering	1	Barter, trade	1	1	BFH-H007
*Ustilago maydis* (DC.) Corda	Huitlacoche	Edible	Maize fields	Gathering	1, 3	Barter, trade	4	2	PhR
*Boletus aestivalis* (Paulet) Fr.	Hongo de pan	Edible	Pine-oak and oak forests	Gathering	3	Barter, trade	2	1	BFH-H008

*Markets studied: *1* Barter Market, *2* Phurépecha Tiánguis, *3* Municipal Market

**Table 3:** Aspects of the process of interchange of the wild and weedy plant resources and wild mushrooms interchanged in the traditional Phurépecha markets of the region of the Pátzcuaro Lake

Aspects	Barter Market	Phurépecha Tiánguis	Municipal Market
Interchange space	Construction based on social relations, offering of products and the custom of interchanging	Construction based on social relations enhancing a space of coexistence, offering products and maintaining the custom of barter	The infrastructure is permanent, people make use of available spaces for developing traditional interchange
Form of exhibiting products	On the floor, on clothes and plastic pieces, baskets, buckets, wooden or plastic boxes, bunched or in plastic bags, separated from cultivated or manufactured products		
Participants	Peasants, gatherers, artisans, fishermen, and women		
	From 29 Phurépecha and Mestizo communities	From 5 Phurépecha and Mestizo communities	From 17 Phurépecha and Mestizo communities
Interchange type	Barter and trade	Barter	Trade
Interchange strategy	Complementing the weekly availability of edible resources for households. Wholesale buying for re-selling in other markets. Moving among markets. Selling to re-sellers	Complementing the weekly availability of edible resources for households. Itinerant interchange.	Wholesale buying for re-selling in other markets. Selling to re-sellers. Buying products to producers and gatherers.
Households members participating in interchange	Mainly women older than 40 years		
Wild and weedy resources interchanged, types of use and management	37 plant species and 15 edible mushroom species; 30 species used as food, 13 medicinal and 9 ceremonial.	15 plant species and 3 edible mushroom species; 11 species used as food, 5 medicinal and 1 ceremonial.	26 plant species and 12 edible mushroom species; 23 species used as food, 9 medicinal and 6 ceremonial.
	Management through gathering strategies, tolerance and enhancing		
Seasonal availability	Throughout the whole year. The highest number of wild and weedy species from June to October. On average, 12 species of wild and weedy resources interchanged per month		Throughout the whole year. On average, 6 species of wild and weedy resources interchanged per month.
Spatial availability	Distributed in forests, agricultural and ruderal areas, riparian vegetation, homegardens		
	From 29 communities of the Pátzcuaro Lake shoreline and the Phurépecha Plateau.	From 5 communities of the Pátzcuaro Lake shoreline.	From 17 communities of the Pátzcuaro Lake shoreline

In the Phurépecha Tiánguis, the most common form of interchange is barter, since the principle of the market is to recover and to maintain the traditional Phurépecha customs, enhancing the mutual help, reciprocity, inter-communitarian relationships, traditional values, and cultural meaning of goods and resources. However, for products of relatively high economic value like tables, chairs, or ceramic, the monetary interchange is allowed after barter is finished in a market day; otherwise, people offering these products difficultly would receive the equivalent amounts of other resources.

In the Barter Market and Phurépecha Tiánguis, barter is carried out through, based on the consideration of the use value, the amount of work invested for producing or obtaining a product, the quality and quantity, seasonality, the need and substitutability of a resource, and in some cases, their monetary value in the markets. In the Phurépecha Tiánguis, other forms of interchange coexist like presents, reciprocity, or mutual help, in the context of inter-personal relations, which may be occasional or highly common.

The commercialization through money is the main form of interchange established in the Municipal Market, where wild and weedy plant resources considered as having high economic value and high demand are offered. The monetary interchange is carried out considered prices, which are firstly established by the sellers but submitted to haggling.

In general, in the three markets, the edible and medicinal wild and weedy plants, as well as mushrooms are interchanged mainly through both barter and trade (37 species, 26 of them edible, 9 medicinal plants, and 2 ornamental plants). Only three wild species with medicinal use are exclusively interchanged through barter, whereas almost all plant species with ceremonial and ornamental uses (11 species) are exclusively traded and considered as those with the highest economic and cultural value.

### Strategies of interchange

Interchange through barter for complementing the weekly requirements of households is the most common strategy of the participants in the Barter Market and the Phurépecha Tiánguis. There, people interchange products gathered, processed, produced by their households, or obtained through interchange with other households in their communities.

There is another strategy of interchange, practiced by traders and wholesalers, who buy great amounts of wild and weedy plants and mushrooms of the region. Then, these traders sell the products in larger markets in the cities of Uruapan, Nahuatzen, Pichátaro, Tacámbaro, and Mexico City. For this strategy, the Barter Market and the Municipal Market are collection centers of the regional plants and mushrooms they commercialize. Through this strategy, great amounts of plants and mushrooms are interchanged exclusively by trading, which reflects the high economic value of those resources and their potential of commercialization in mercantile contexts. This strategy of interchange involves fruits of capulín (*Prunus serotina*), zarzamora (*Rubus liebmanii*), all mushrooms species recorded, anís (*Tagetes micrantha*), quelite cenizo (*Chenopodium berlandieri*), and árnica (*Heteroteca inuloides*).

Some people used to go to the Barter Market in the morning and to the Municipal Market in the afternoon, alternating barter and monetary interchange types, respectively. This strategy is practiced mainly by people that carry large amounts of products, or products with high economic value, or products that are offered during short time periods. This strategy looks for broaden possibilities of interchange. These are for instance the cases of sellers of bunches of the flowers called “estrellitas” (*Milla biflora*) and orchids for rituals (*Laelia autumnalis* and *L. speciosa)*. Also, these are the cases of sellers of cladodes of *Opuntia atropes* which are consumed as greens, fruits of *Rubus Liebmannii*, and bunches of “anís” (*Tagetes micrantha*), a species that is widely used as flavoring (Tables 3 and 4).

**Table 4:** Aspects of the complex *Kosmos-corpus-praxis* of wild and weedy resources interchanged in traditional markets of the Phurépecha region

Group of resources	Scientific name	Common name	Aspects of the complex *Kosmos-corpus-praxis* of wild and weedy resources interchanged in traditional Phurépecha markets
Quelites and opuntia cladodes	*Amaranthus hybridus* L.	Quelite de trigo, quintonil	For Phurépecha people, this group of plants represents food of good quality, clean, free of agrochemical products, and nutritious. Considered of great importance in people’s life since become basic food in particular seasons of the year. Plants appreciated as traditional Phurépecha food, providing notion of belonging to the Phurépecha culture, remaining in the memory as food consumed by ancient people and those participating in the markets since they were children. Valued as indispensable in household subsistence, food, and interchange value. There are traditional ecological knowledge generated and transmitted about seasonality, distribution forms of propagation, among the most relevant, in addition to gastronomic knowledge about preparation, consumption and nutritious qualities. Management practices are simple gathering of juvenile plants in forests and agricultural areas; tolerance (let standing during perturbation) and enhancing through propagating seeds and seedlings.
	*Brassica rapa* L.	Mostaza	
	*Chenopodium berlandieri* Moq.	Quelite cenizo	
	*Opuntia atropes* Rose	Nopales	
	*Portulaca oleracea* L.	Verdolaga	
	*Rorippa nasturtium-aquaticum (*L.) Hayek	Berro	
	*Rumex obtusifolius* L.	Juan primero	
Fruits and stems	*Agave inaequidens*	Jiote	These are food of excellent quality, clean because they are wild. Considered as fruit belonging to Phurépecha people. Resources of high importance in people’s life, as complementary food in particular seasons. Highly appreciated as traditional food of the Phurépecha culture, found in the memory of food consumed by ancient people. Valued since they complement household’s subsistence, have commercial value for obtaining other products through interchange. Local people appreciate these fruits as part of the Phurépecha diet, good flavor, and high nutritious and medicinal properties. Traditional ecological knowledge about life cycle, distribution, seasonality, sexual and asexual propagation, and transplanting success has been generated and transmitted. In addition, knowledge about forms of preparation, nutritious and medicinal properties was recorded. Management practices are used, among them are simple and selective gathering in wild populations, identifying and differentiating varieties of fruits in some species, tolerance, transplanting and propagation in agricultural areas and homegardens.
	*Crataegus mexicana* Moc. & Sessé ex DC	Tejocote	
	*Opuntia* sp.	Xoconostle	
	*Prunus serotina* subsp. *capuli* (Cav. ex Spreng.) McVaugh	Capulines	
	*Rubus Liebmannii* Focke	Fruto de zarzamora	
	*Solanum lycopersicum* L.	Jitomate silvestre	
Flavorings	*Tagetes micrantha* Cav.	Anís	This group of plants is highly appreciated since improve flavor of food and because has medicinal properties. These plants are considered part of the Phurépecha communities. These species are part of Phurépecha people’s life providing flavoring for food and traditional beverages consumed in the daily life and ceremonies; these are also appreciated as providing feeling of belonging to the Phurépecha culture. These plants are valued as supporters of the households’ subsistence because of their interchange value since they are highly required for preparing food. Traditional ecological knowledge was documented about morphology, seasonality, distribution, mechanisms of propagation, as well as traditional gastronomic recipes, nutritious qualities and medicinal properties. Management practices on these plants include simple gathering from wild populations, tolerance, transplanting of juvenile plants, propagating them in agricultural areas and homegardens. Their management is considered an activity conducted by women.
	*Dysphania ambrosioides (*L.) Mosyakin & Clemants	Epazote	
Medicinal	*Acalypha phleoides* Cav.	Hierba del cáncer	People confer to these plants the meaning of natural medicine, and are highly appreciated as part of the Phurépecha medicine. Contribute to alleviate physic and spiritual pains, and are part of the religious ceremonies, Phurépecha rituals and customs. These plants are considered as heritage of their ancient Phurépecha relatives, having edible and commercial value. Traditional ecological knowledge was recorded in relation to distribution, seasonality, particularly of useful parts, and this information is transmitted to new generations. In addition, people recognize their medicinal properties, pains that are alleviated, forms of use and doses, as well as forms of conserving them. Management practices include gathering in agricultural and ruderal areas, riparian vegetation and forests. People enhance their abundance by propagating them (by women) in homegardens. In addition, people procure their availability though dehydration.
	*Agastache mexicana* (Kunth) Lint & Epling	Toronjil	
	*Artemisia ludoviciana* Nutt.	Istafiate	
	*Chenopodium graveolens* Lag & Rodr.	Epazote de perro	
	*Clinopodium macrostemum* (Moc. & Sessé ex Benth.) Kuntze (*Satureja macrostema* (Benth.) Briq.)	Nurite	
	*Equisetum* sp.	Cola de caballo	
	*Eryngium carlinae* F. Delaroche	Hierba del sapo	
	*Gnaphalium* spp.	Gordolobo	
	*Heterotheca inuloides* Cass.	Árnica	
	*Loeselia mexicana* (Lam.) Brand	Espinosilla	
	*Marrubium vulgare* L.	Marubio	
	*Ternstroemia lineata* DC.	Trompillo	
Ceremonial-ornamental	*Laelia autumnalis* (Lex.) Lindl.	Flor de ánima o lirio	For Phurépecha people, flowers represent beauty, the ornaments and luxury; represent also the link and communication with the sacred world and with dead people. In Phurépecha, these plants are grouped in the category “*ambakiti*”. Flowers are highly appreciated and considered indispensable as part of the ceremonial and religious life. Ecological knowledge was recorded in relation to seasonality, distribution, abundance, interactions, their sexual and asexual propagation and responses to transplanting. Management practices include gathering from wild populations, tolerance, and propagation in agricultural areas and homegardens. This latter is recognized as an activity practiced by women.
	*Bryophyta* sensu *lato*	Musgo	
	*Calochortus purpureus* (Kunth) Baker	Flores moraditas	
	*Castilleja scorzonerifolia* Kunth	Flor de terciopelo	
	*Cosmos bipinnatus* Cav.	Mirasoles	
	*Laelia speciosa* (Kunth) Schltr.	Orquídea, flor de *corpus*	
	*Lupinus montanus* Kunth	Flor morada	
	*Stevia monardifolia* Kunth	Servilletilla	
	*Tillandsia* sp.	Heno	
	*Milla biflora* Cav.	Estrellitas	
	*Tagetes lucida* Cav.	Santa María	
Mushrooms	*Ramaria fenica* (P. Karst.) Ricken	Patitas de pájaro	Wild edible mushrooms are considered food of high quality, flavor, clean, and nutritious (their properties considered better than cattle and pig meat). Some species are considered as luxury food. Mushrooms are resources of great importance in people’s life, as basic food during the seasons when these are available, the rainy season. Provide the feeling of belonging to the Phurépecha culture and are part of the memory of food consumed by ancient people. Are highly valued in the interchange and, therefore, highly valued by people as the means for obtaining other products. Mushrooms are part of a wide variety of traditional food, particularly the scarce species are considered as luxury food. Traditional ecological knowledge is particularly important for recognizing the edible and non-edible species. People know about their properties, their seasonality, areas of distribution, forms of preparation, and consumption. Mushrooms are gathered mainly in areas of pine-oak and oak forests and in grasslands, mainly, by men and, occasionally, by women.
	*Ramaria flavigelatinosa* Marr & D.E. Stuntz	Patitas de pájaro	
	*Ramaria araiospora* Marr & D.E. Stuntz	Patitas de pájaro	
	*Ramaria botrytis* (Pers.) Ricken	Patitas de pájaro	
	*Ramaria flava* (Schaeff.) Quél.	Patitas de pájaro	
	*Lyophyllum connatum* (Schumach.) Singer	Guachitas, pashacuas	
	*Lyophyllum decastes* (Fr.) Singer	Guachitas, pashacuas	
	*Agaricus campestris* L.	Hongo llanero	
	*Amanita caesarea* (Scop.) Pers.	Hongo amarillo	
	*Hypomyces lactifluorum* (Schwein.) Tul. & C. Tul.	Hongo trompa de puerco	
	*Calvatia cytahiformis* (Bosc) Morgan	Hongo globoso	
	*Helvella crispa* (Scop.) Fr.	Oreja de ratón blanca	
	*Laccaria laccata* (Scop.) Cooke	Moradito	
	*Ustilago maydis (*DC.) Corda	Huitlacoche	

Another strategy of interchange is that of the participants in the Barter Market, who establish relationships with sellers of the Municipal Market and other merchants of the city of Pátzcuaro to whom people bring products “por encargo” (especially in charged). For instance, a family of Chapultepec, Michoacán offers in the Barter Market a broad spectrum of wild medicinal and edible plants, but sell to merchants of the Municipal Market, while other informal sellers of Pátzcuaro sell specific in charged plants like “berros” (*Rorippa nasturtium-aquaticum*), “cola de caballo” (*Equisetum* sp.), and “árnica” (*Heterotheca inuloides*). Families of San Pedro Pareo and Ihuatzio are specifically in charged to bring large amounts of “quelite cenizo” (*Chenopodium berlandieri*) to wholesalers who in turn sell these plants in Nahuatzen and Uruapan. But this strategy involves exclusive trading.

The strategy of itinerant interchange practiced through the Phurépecha Tiánguis enhances interchange of products particularly abundant or specifically available in the visited community. Some of the participants in this Tiánguis interchange large amounts of products offered by sellers of the visited community, in order to store and interchange them in other markets or in other communities participating in the “Phurépecha Tiánguis”. In addition, the strategy of the participants in this Tiánguis is to offer products highly demanded by the visited communities, for instance, wooden tools demanded by the communities close to the lake, which have low access to wood. The participants of the Tiánguis consider this market as an opportunity to offer their products while obtaining others.

To the Municipal Market arrive producers of rural communities of the region offering products from agriculture and gathering. They do not have an established place in the market and look for sites available for offering their products, and if no place is available, they look for an alternative place in the Municipal Market, in the Barter Market or in the streets of the city of Pátzcuaro. Among the products offered through this strategy, we recorded “anís” (*Tagetes micrantha*), “estrellitas” flowers (*Milla biflora*), orchids (*Laelia autumnalis* and *L. speciosa*), cladodes of *Opuntia atropes*, several species of mushrooms (mainly *Agaricus campestris*, *Amanita caesarea*, *Hypomyces lactiflorum*, and *Ramaria* spp.), “quelite cenizo” (*Chenopodium berlandieri)*, “mostaza” (*Brassica rapa*), “quelite de trigo” (*A. hybridus)*, “verdolagas” (*Portulaca oleracea*), and “berros” (*R. nasturtium-aquaticum*).

Some formal and informal sellers of the Municipal Market broaden their offer of products by including plants and mushrooms they directly collect or buy from gatherers of the Phurépecha and Mestizo communities. For instance, gatherers of edible mushrooms of the communities of Yotatiro and La Zarzamora sell mushrooms to sellers from the Municipal Market. Among the wild and weedy plants offered through this strategy, we recorded the “estrellitas” flowers (*M. biflora*), “mirasoles” (*Cosmos bipinnatus*), and orchids (*L. autumnalis*), as well as several species of mushrooms (mainly *Agaricus campestris*, *Amanita caesarea*, *Hypomyces lactiflorum*, and *Ramaria* spp.), “heno” (*Tillandsia usneoides*), and “musgos” (Bryophyta sensu *lato*).

### Households’ members participating in interchange

In the Barter Market and Phurépecha Tiánguis, women older than 40 years are those mainly carrying out the interchange, although they may go to the market with other members of the family, they are the ones who carry out the interchange. Mainly because they are the responsible of guaranteeing the weekly availability of products for the households; therefore, they know what products, how much, and which type and quality of them their households need. Most of the participants in these markets are women, accompanied mainly by children, who learn through these experiences the art of interchanging and bartering. The Phurépecha Tiánguis uses to dedicate the first 15 min of activity to the participation of children in bartering in order to teach to them the activity of interchange. In the Municipal Market, the sellers are women who exhibit and interchange their products, and only in one occasion had we recorded a man commercializing wild mushrooms.

### Wild and weedy plants and mushrooms interchanged, their use, and management

**Fig. 3:**
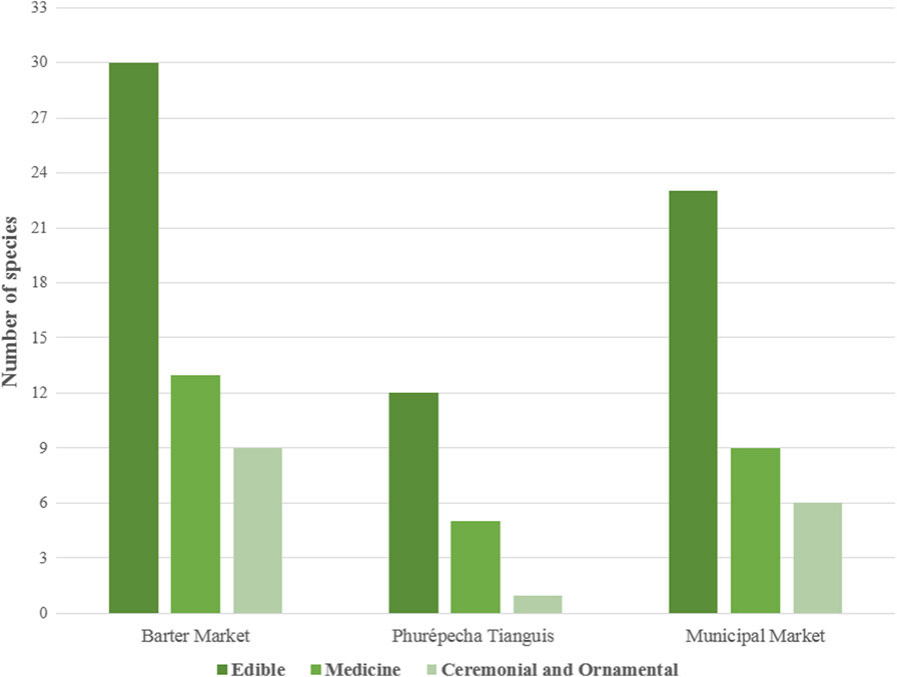
Number of plant species and mushrooms edible, medicinal, ceremonial, and ornamental interchanged in the Barter Market, the Phurépecha Tiánguis, and the Municipal Market in the Pátzcuaro Lake and the Phurépecha Plateau in Michoacán, Central Mexico

We recorded 53 species of wild plants and mushrooms and weedy plants interchanged in the traditional markets studied. In the Barter Market, we recorded 85.5% of these resources, 37 species of plants, and 15 species of mushrooms. The products offered in the Barter Market are food, traditional medicine, products for ritual ceremonies, and ornaments for the Phurépecha communities. In the Barter Market, we recorded 30 wild and weedy species used as food, 13 used as medicine, and 9 used for ritual ceremonies and ornaments (Fig. 3). These resources are extracted from forests, agricultural and ruderal areas, and homegardens, where people gather, tolerate, and enhance most of the interchanged plant species. We in addition recorded processed products like cooked escapes of agave (*Agave inaequidens*), tamales prepared with wild *Rubus liebmanii*, cooked cladodes of *Opuntia atropes*, and baskets weaved with “chuspata” and “tule” (*Typha* sp. y *Schoenoplectus* sp., respectively), which are aquatic plants extracted from the lake. Wild mushrooms are extracted from forest areas and grasslands in the territories of the communities close to the lake basin, as well as from other more distant communities like Cuanajo, Pichátaro, and Zirahuén.

In the Phurépecha Tiánguis, we recorded the interchange of 34.5% of wild and weedy resources registered in the study, 15 species of plants and 3 species of mushrooms. The wild and weedy resources interchanged are part of the food (11 species), traditional medicine (5 species), and ceremonial practices (1 species) of the communities (Fig. 3). These resources are extracted from forests, agricultural and ruderal areas, and homegardens (gathered, tolerated, and enhanced in anthropic areas). In addition, some processed products are offered, as these are the cases of tamales prepared with *Rubus liebmanii*, jams prepared with dehydrated tejocotes (*Crataegus mexicana*), cooked cladodes of *Opuntia atropes*, and baskets of *Typha* sp. and *Schoenoplectus* sp. Wild mushrooms are extracted from pine-oak forests from Cuanajo and Santa Fe de la Laguna.

In the Municipal Market, we recorded the interchange of 69.1% of the wild and weedy resources registered, 26 species of plants and 12 species of mushrooms, and 23 species are used as food, 9 as medicine, and 6 as ceremonial and ornamental (Fig. 3).

Most edible plants and mushrooms recorded (30 species) are obtained through gathering, but tolerance and enhancing through sowing and plating are practiced on 5 of these species. All wild and weedy medicinal plants recorded (14 species) are obtained through gathering and propagation (or enhancing), whereas all wild plants recorded (11 species) for ceremonial and ornamental use and mushrooms are exclusively gathered in the wild.

### Seasonal and spatial availability of wild and weedy resources in the markets

Throughout the whole year, there is offering of wild and weedy resources in the three markets studied, satisfying the basic needs of food, medicine, ornamental, and ritual requirements of the regional people of the Pátzcuaro Lake and Phurépecha Plateau areas. However, some particular resources are available during particular periods.

The traditional greens or “quelites” Juan Primero (*Rumex obtusifolius*), the “quelite cenizo” (*C. berlandieri*), and “berros” (*R. nasturtium-aquaticum*) are available throughout the whole year because these are weedy plants growing in seasonal and irrigated agricultural systems. The “quelites de trigo” (*Amaranthus hybridus*) and the “verdolagas” (*P. oleracea*) are available during the rainy season. The cladodes of the wild *Opuntia atropes* are abundantly offered from January to August. Wild fruits like the blackberries (*Rubus liebmannii*) are intermittently available from February to September, whereas fruits of the “capulín” (*Prunus serotina*) from May to July. The epazote (*Dysphania ambrosioides*) and “anís” (*Tagetes micrantha*) used as condiments and flavoring are offered mainly during the rainy season. Much wild plants used as medicine are offered 8 to 11 months per year; these are the cases of “árnica” (*Heterotheca inuloides*), “cola de caballo” (*Equisetum* sp.), and “manrubio” (*Marrubium vulgare*). Other medicinal plants are available 4 to 6 months per year, as it is the cases of “toronjil” (*Agastache mexicana*), “nurite” (*Clinopodium macrostemum*), and “gordolobo” (*Gnaphalium* spp.); others that have lower cultural and economic importance are available in the markets 3 months per year (Tables 1, 2, and 3).

The offering period of wild flowers for ritual and ornamental purposes are determined by their flowering seasons, and these are coupled with specific ceremonies and rituals; for instance, the flower of *Corpus Christi* (*Laelia speciosa*) is available from May to June and used for the offerings and ornaments dedicated to the celebration of *Corpus Christi*. The flower of the “ánima” (*Laelia autumnalis*) available in October and November is used for the offerings of the Day of the Dead; other species like *M. biflora*, *Castilleja scorzonerifolia*, *Cosmos bipinnatus*, and *Tagetes lucida* are utilized as offerings to saints and temples in homes and the communities. Wild mushrooms are abundant from the end of June to September, some years even in October and November, when the rainy season becomes prolonged.

**Fig. 4:**
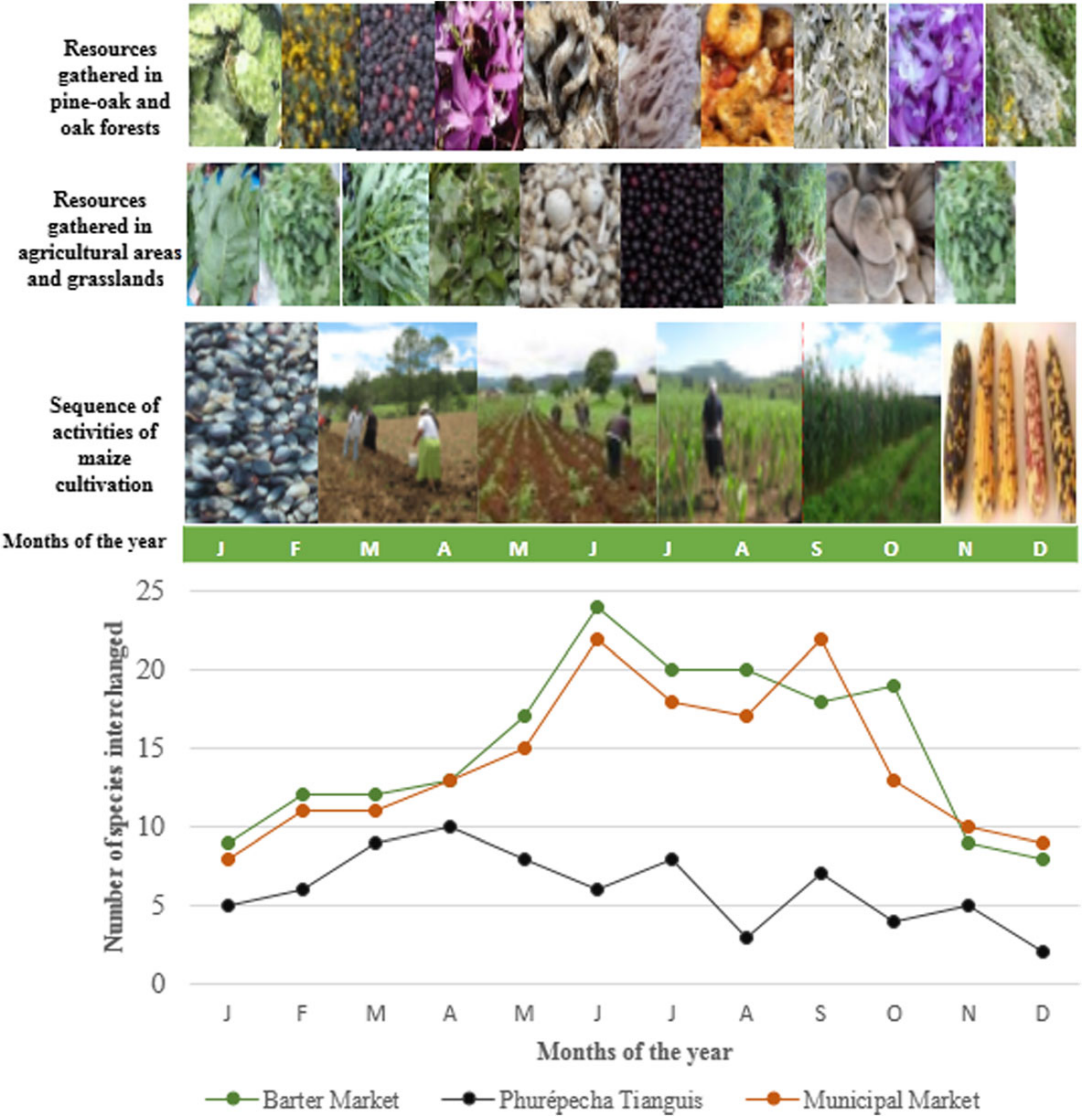
Aspects of the wild and weedy resources gathered, tolerated, and enhanced in pine-oak and oak forests, agricultural and grassland areas, and a sequence of the main activities of maize agriculture. The plot illustrated the availability (total number) of wild and weedy products in the Markets studied throughout the year

The diversity of products offered in the markets is related with the environmental variability of the Pátzcuaro Lake and the Phurépecha Plateau, whose landscape is conformed by areas of aquatic vegetation, plain valleys used for agriculture, grasslands used for livestock raising, forest areas with subtropical scrubs, pine, pine-oak, and oak forests (Fig. 4).

Some wild and weedy resources are offered by a large number of communities, which reflects their broad spatial availability, as well as their high demand by regional people. The greens “quelites” are offered by 16 communities from the shoreline of the Pátzcuaro Lake and are produced in seasonal and irrigated agricultural plots. In the Phurépecha Plateau, quelites are produced in seasonal agriculture plots. Cladodes of *Opuntia atropes* are offered by 13 communities of the area of subtropical scrub in the shoreline of the Pátzcuaro Lake. Plants used a condiments and flavoring (mainly “anís” and “epazote”) are greatly appreciated by regional people and are offered by 12 communities from the Pátzcuaro Lake and the Phurépecha Plateau. Medicinal plants are offered by 11 communities whereas edible mushrooms by 9 communities, mainly from the Phurépecha Plateau. The wild ornamental flowers and fruits are offered by seven communities, which extract them from agricultural areas, grasslands, forest areas of subtropical scrub, pine, pine-oak, and oak forests.

Edible and medicinal plants and mushrooms recorded are obtained from forests, ruderal areas, agricultural plots, and homegardens (as weeds) and from the aquatic vegetation in the lake. Plants with ceremonial and ornamental use are extracted from forests and weedy environments.

### Interchange intensity, pressure, risk, and management of resources interchanged

Wild and weedy plants with the highest cultural and economic values are those interchanged in the highest amounts by the highest number of people, and more frequently, these are the quelite cenizo (*Chenopodium berlandieri*), fruits of capulín *(Prunus serotina*), fruits of zarzamora (*Rubus liebmanii*), anís (*Tagetes micrantha*), árnica (*Heteroteca inuloides*), prickly pear cladodes (*O. atropes*), and the mushrooms *Ramaria* spp., *Hypomyces lactifluorum*, *Lyophyllum* spp., and *Amanita* spp. Although the spatial and temporal availability of these species is highly variable, people put in practice management strategies in order to ensure their availability and needed abundance. For instance, *C. berlandieri* is under simple gathering, but in addition, people use to disperse seeds in order to increase their abundance in seasonal and irrigated agricultural plots where they grow as weeds, having them available throughout the year. In the case of *Prunus serotina*, people use to selectively harvest large purple fruits and selectively promote tolerance and transplanting of trees with these attributes in homegardens and other agroforestry systems. These strategies are motivated by traditions associated to direct consumption, but also to bring fruits to spaces of interchange.

In the cases of mushrooms with high cultural and economic value and interchange intensity, we could not identify specific management practices. People mentioned that some species like *Hypomyces lactifluorum* are progressively scarcer, and such scarcity is attributed to the extraction of ground in forests (which is also commercialized in cities for cultivating ornamental plants), as well as to deforestation, and some communities have started to establish in relation to these practices, which affect not only the availability of mushrooms.

We identified some species seasonally interchanged due to their short-time period availability, but whose availability are in risk since their forms of use and amounts extracted are high. This is the case of *Laelia speciosa*, which is extracted completely (both bulbs and flowers), and there are no practices of management in order to promote its recovering or increasing its abundance. The species *L. autumnalis* is intensely extracted through simple gathering for a period no longer than 15 days which similarly to *L. speciosa* determines high risk not attended through management practices. The flowers of *Milla biflora* are extracted intensely during 1 or 2 months, and this activity along with other changes in land use have contributed to their scarcity, determining that people have to go progressively farer away to collect them. However, neither practices of management nor regulations were recorded, at least from interviews in markets.

Some medicinal plants like *Clinopodium macrostemum* and *Agastache mexicana* have high cultural importance and demand in the markets. Although amounts interchanged every time are relatively low, it occurs with high frequency. These species are under simple gathering from the wild, but some practices like seed sowing and transplanting from forests to homegardens are carried out. However, in the case of *C. macrostemum*, plants managed are not appreciated since according to people these do not have the smelling and flavor of those from the wild. These plants occur in specific environments and gathering them already represent risk, which is not satisfactorily attended yet. The árnica (*Heterotheca inuloides*) is also intensely extracted through simple gathering with no management practices. Local people consider that management is unnecessary since it is an abundant plant without risk.

## Discussion

The traditional markets studied significantly contribute to maintain biodiversity, human culture, and social relations in the Phurépecha region, expressing main cultural values, customs, and life strategies, in relation to knowledge and management of the interchanged resources [17, 18, 29–35]. These markets represent the relation of humans with biodiversity through TEK and management practices [18, 36]. The space of interchange in the traditional markets studied is generated through social relations constructed based on products that are valued within the perspective of a culture of food, medicine, ceremonies, and forms of life that are part of the Phurépecha identity [22, 32], which enhance the diversified use of products through spaces of convergence of people from different communities [5, 19, 20, 22]. Women are the main connectors of the households’ world (what they produce, gather, process, and/or manufacture) with other households [20].

The form of offering products in traditional markets is similar to that described in pre-Columbian historical sources [37], which represents a cultural continuity [25, 38]. The possibility of interchanging a product is socially relevant, and it depends on the features of that product’s interchangeability within a cultural context. Things and products are classified and valued as a function of their meaning, values, communitarian regulations, and the practices of interchange themselves. Therefore, the interchange is a main source of referents for valuing products in a particular cultural context [5, 19, 32, 39, 40]. Interchange is a basic aspect of the subsistence strategy of households of the Pátzcuaro Lake region, which makes it possible to obtain products from different ecological regions [16, 19–21].

Bartering is an ongoing form of Mesoamerican interchange. Through barter, horizontal social relations are established among people practicing it, and the notion of belonging to a cultural group is affirmed, when sharing products as a series of codes learned and shared characteristics of a human culture [32, 40]. In addition, the participants in bartering transactions identified themselves with things and products interchanged according to their use value, meaning, equivalence, prestige, need, and desire, which are all considerations based on a general worldview. The wild and weedy plants and mushrooms interchanged are mostly basic and complementary food for the regional people and considered of high economic and cultural importance. In the markets described, barter is maintained as a tradition and as part of a subsistence strategy for interchanging products in contexts in which money has still low importance. Barter is a node of a net of actions associated to management of natural resources for satisfying the subsistence needs of households that obtain products, constructing relations of reciprocity, solidarity, trusting, and equity [26].

The spaces of interchange are settings for developing a great variety of strategies of interchange that remain in the cultural memory. Persons participating in markets produce use values for other persons through a high variety of management strategies [39]. The wild and weedy resources interchanged in the region studied are mostly native species of plants and mushrooms that historically have high cultural value [20]. Gathering of these resources are carried out together with agricultural and forestry practices at household and regional levels (Fig. 4). Most wild and weedy species are available when availability of maize is low, when people is waiting for the products of the new harvest, and by that time, wild and weedy resources are particularly important for household subsistence. The markets therefore reflect the still cultural importance of gathering and extraction of wild and weedy products in people subsistence.

People practice management of wild and weedy plant resources, and this management is influenced by their role in subsistence, which in turn influence their exchange value. This value is therefore an important indicator of the motivation of people to gather and manage the resources interchanged, in order to increase their availability whenever it is necessary (Table 4).

The value of a product is not inherent to the product, but a property given to it by people. The use value make reference to the utilitarianism of a product for a specific cultural group while the interchange value is the capacity of a product for obtaining other products in a context of mercantile or not mercantile interchange [11, 22, 32, 39, 41, 42]. The wild and weedy resources interchanged in the markets studied are valued by the properties, qualities, and meanings assigned by the Phurépecha people [20, 26]. The value of a product is therefore the result of its position and meaning in a worldview (*kosmos*) of a human group, which involves a universe of incommensurable symbolic aspects. It is also related to the knowledge about properties and qualities (*corpus*), and the effort invested for making it available through practices (*praxis*) (Table 4).

Barter and trade represent different forms of interchange, which involve different forms of transactions and amounts of resources. Barter generally involves a higher diversity of products and species interchanged in small amounts, whereas trade may involve great amounts of products that are carried from the Phurépecha region to other areas. These transaction relations determine differential pressures on resources.

Some wild and especially weedy plants (some edible greens called *xakua*) are enhanced throughout the year in agricultural areas with irrigation. These species are therefore continually available and in appropriate amounts. Contrastingly, some products with high cultural and economic value, like ceremonial and ornamental plants, are exclusively gathered from the wild and no management was recorded. These species, although with high cultural value are required in smaller amounts than those managed to ensure greater amounts. Estimating with precision the amounts of products interchanged is particularly difficult in traditional markets which are highly dynamic in people and products occurring there throughout the year. But a strategy for an evaluation about this issue would provide more precise information to be contrasted with their regional availability.

This study allows establishing some general relations between demand, perception of risk, and responses of management in relation to that risk. From markets, interviews and qualitative information about what people perceive provide valuable information in this respect. We identified groups of resources that are highly demanded in markets, that are actively interchanged throughout the year, and that receive management. We identified simple gathering, selective gathering, and gathering involving low or high amounts of time invested to obtain them, together with specific tools. Gathering is, therefore, not necessarily a simple action. Along with gathering, a higher complexity can be identified in tolerance or let standing of wild plants in anthropogenic areas, and such tolerance can be selective. Similarly, people promote or enhance plants, some of them selectively, and some others may be sowed and transplanted. All these practices represent a gradient of forms of interactions between people and plants, and some of them with mushrooms. These interactions and relations are similar to those documented by our research group in the Tehuacan Valley [11, 13, 20].

It is particularly relevant to say that for the moment, we have identified clear relations of these interactions as responses to risk in most of the species studied, but not in all of them. For instance, those medicinal plants whose properties are considered to be lost through management, or those ornamental plants like *Laelia* spp. whose management may be difficult, or in general, lack of management strategies of mushrooms offer important challenges to continue analyzing motivations and limitations to management of biotic resources. Interchange may be the cause of risk for some species where these are under higher pressure, and risk may be a primary motivation to management. However, perception of risk may be highly heterogeneous among people who interact with those biotic resources and these possibilities of carrying out management or innovations may have some limitations associated to biological aspects of the resources. For instance, seed germination, transplanting, or survival of managed resources could be unsuccessful and more specific techniques needed. The analysis of these relations from the spaces of interchange is an important door for understanding them. Markets allow documenting what people perceive, but more specific studies from the villages where the resources occur and are extracted and managed are a crucial complement.

The Phurépecha markets are valuable settings of culture, social relations, and technology to manage both biotic resources and ecosystems. Important lessons for understanding factors motivating management can be found in these contexts, as well as in connection with the settings of the management of the territories of the communities participating in the net of interchange of the region. However, deeper information is still needed from people and communities where products destined to markets occur and are used, and management by people of those settings, the territories of the villages, is the topic of our ongoing studies.

## Conclusions

The wild and weedy plants and mushrooms interchanged in the traditional markets of the Phurépecha region are key cultural species, immersed in the local cultural traditions of food, health, religious ceremonies, and rituals for daily life [43–47], clearly representing aspects of the worldview, knowledge, and practices of the Phurépecha people. These aspects deserve an ethnoecological approach of the world around these resources and the relations of interchange involving them.

The traditional markets are part of a strategy of subsistence of Phurépecha people of the region, which conform an important setting of social relations, interchange of products, and cultural identity, and a crucial context for conserving the Phurépecha worldview, knowledge, and management practices [20, 25, 32]. The markets are therefore crucial expression of ethnoecological dimensions of the Phurépecha people.

Wild and weedy plants and mushrooms interchanged are important edible, medicinal, and ceremonial resources, mainly obtained through gathering from forests and weedy environments, but some of them, particularly edible and mainly medicinal plants, are managed through enhancing them in human-made environments. In general, the demand of products in markets enhances innovation and practices for ensuring or increasing their availability, particularly those that are naturally scarce. It was notorious that, with the exception of most, mushrooms and ritual plants have high demand and value in markets, but all wild resources for satisfying these needs are obtained through simple gathering.

## Abbreviation

UNAM: Universidad Nacional Autónoma de México
